# The Greatly Under-Represented Role of Smooth Muscle Cells in Atherosclerosis

**DOI:** 10.1007/s11883-023-01145-8

**Published:** 2023-09-04

**Authors:** Gordon A. Francis

**Affiliations:** Centre for Heart Lung Innovation, Providence Research, St. Paul’s Hospital, University of British Columbia, Vancouver, Canada

**Keywords:** Smooth muscle, Atherosclerosis, Macrophage, Diffuse intimal thickening, Inflammation, Pathogenesis

## Abstract

**Purpose of Review:**

This article summarizes previous and recent research on the fundamental role of arterial smooth muscle cells (SMCs) as drivers of initial and, along with macrophages, later stages of human atherosclerosis.

**Recent Findings:**

Studies using human tissues and SMC lineage-tracing mice have reinforced earlier observations that SMCs drive initial atherogenesis in humans and contribute a multitude of phenotypes including foam cell formation hitherto attributed primarily to macrophages in atherosclerosis.

**Summary:**

Arterial smooth muscle cells (SMCs) are the primary cell type in human pre-atherosclerotic intima and are responsible for the retention of lipoproteins that drive the development of atherosclerosis. Despite this, images of atherogenesis still depict the process as initially devoid of SMCs, primarily macrophage driven, and indicate only relatively minor roles such as fibrous cap formation to intimal SMCs. This review summarizes historical and recent observations regarding the importance of SMCs in the formation of a pre-atherosclerotic intima, initial and later foam cell formation, and the phenotypic changes that give rise to multiple different roles for SMCs in human and mouse lesions. Potential SMC-specific therapies in atherosclerosis are presented.

## Introduction

Atherosclerosis is the underlying illness resulting in more mortality than any other cause internationally [1]. Despite over 150 years of research on its pathogenesis, controversies still remain regarding the initial steps in the disease process and the main cell types mediating lesion development. Arterial smooth muscle cells (SMCs) are estimated to make up > 90% of all cells in human atherosclerotic plaque [2] and are present in pre-atherosclerotic arterial intima starting in utero [3]. Despite this, the majority of images of developing and advanced atherosclerosis in major reviews of the pathogenesis of this disease continue to focus primarily on the role of macrophages and to depict SMCs as having only a relatively minor role in the disease usually confined to how they form the fibrous cap over lesions. This article reviews key historical observations about the cellular makeup of human plaque and the basis for the response to retention and response to injury hypotheses of atherogenesis. Recent studies on the multiple roles of SMCs in plaque and the potential for therapies that alter SMC phenotype to make further advances in the prevention and treatment of atherosclerosis are highlighted.

### Historical Reports of Early Stages of Human Atherogenesis

**Fig. 1:**
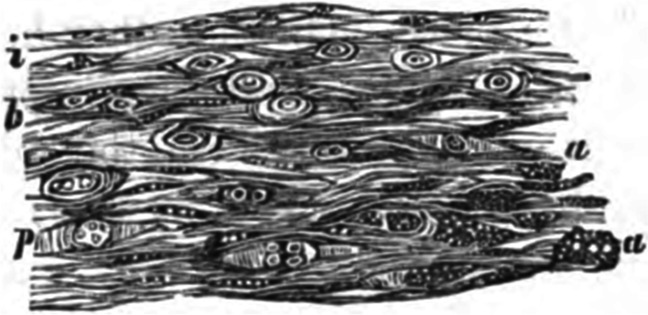
Drawing by Virchow of a vertical section of atherosclerotic aortic plaque. (a) Spindle-shaped atheromatous foam cells in deep intima; (p) proliferating cells with evidence of dividing nuclei; (b) layer of cells beneath intact endothelial monolayer (i) on luminal surface. (From Virchow) [4]

Rudolf Virchow, in his cellular pathology lectures in 1858, provided an image of what he termed an early sclerotic plate from human aorta, showing spindle-shaped cells containing lipid droplets and “fatty degeneration” in the deep intima (Fig. 1). The location and morphology of these deep intimal lipid-loaded cells are most consistent with them being SMC foam cells [4].

Rudolf Atschul, also using light microscopy, reported in 1950 that foam cells may arise from smooth muscle [5]. In 1961, Geer et al. provided early electron microscopic images of pre-atherosclerotic thickening of the intima in grossly normal human aorta and coronary arteries and concluded that it consists mostly of endothelium, SMCs and elastic fibers, and only rare histiocytes (resident tissue macrophages or dendritic cells) [6]. They emphasized this musculo-elastic intima to be different from the intima of animal arteries, which lack infiltration of the intima with SMCs and contained only endothelium, a small amount of collagen, and an internal elastic lamina [6]. In the same paper, they provided images of early fatty streaks and made the observations that “the lipid lay almost exclusively in smooth muscle”; “lipid in the more advanced fatty streak often appeared in smooth muscle and foam cells”; and “smooth muscle cells often contained so many vacuoles that they appeared to be in a transitional stage between smooth muscle and foam cells” [6]. In this way, the authors seemed to differentiate a SMC foam cell from other foam cells, but overall concluded the majority of fatty streak lipids were in SMCs, and in both SMCs and macrophages in later lesions.

In 1985, Aqel et al. reported that the cells in diffuse intimal thickening of human atherosclerotic plaques were almost all SMCs, but there were rare subendothelial macrophages. They concluded that the first foam cells found in fatty streaks were macrophage in origin; however, the lipid staining technique shown suggested primarily extracellular lipids around rather than inside macrophages [7]. Wissler in 1991, examining samples from the Pathological Determinants of Atherosclerosis in Youth (PDAY) study, stated “The major components of atherosclerotic plaque are deposited lipids – mostly cholesteryl esters and cholesterol, derived largely from the lower-density lipoproteins of the blood- and proliferated, modified arterial smooth muscle cells with their synthesized connective tissue products” [2]. He further stated “the quantitative microscopic findings suggest many fewer monocyte-derived macrophages in lesions in young people than some reports from less systematic studies of more advanced arterial lesions in older individuals would indicate” [8]. He also concluded from these studies that SMCs “make up 90-95% of the cellular population of the lesion and which, with the synthetic products of these cells, compose an average of 50% of the advanced atherosclerotic plaque” [2].

Katsuda along with coauthors including Ross and Gown reported in 1992, using a comprehensive panel of monoclonal antibodies, that in early fatty streaks in individuals ranging in age 15–34 “the predominant cell type was the smooth muscle cell, including the vast majority of foam cells, which tended to appear in the deeper regions of the lesions” [9].

Ross and Glomset in 1973 considered development of a SMC-rich intima in human arteries to be a later phenomenon not seen in children or young adults that the first phase of a developing atherosclerotic lesion is a focal thickening of the intima consisting of an increase in SMCs and extracellular matrix and that fatty streaks contained lipid deposits mainly within smooth muscle cells, which are surrounded by extracellular lipid deposits [10]. They argued that accumulation of SMCs was necessary for lipid deposition because “the lipid deposits occur either within smooth muscle cells or outside them in association with connective tissue matrix components which are secretory products of smooth muscle cells” [10]. They also proposed that the migration and proliferation of SMCs within the intima and the infiltration of circulating lipoproteins required an initial and sustained injury to the endothelium to result in an enlarging lesion. In their 1976 paper, they formally proposed the response to injury hypothesis of atherosclerosis pathogenesis [11]. This theory was based on what they considered the similarities between atherosclerosis and the response of arteries to experimental injury in animal models. It relies on there being injurious factors that can cause focal detachment of endothelial cells to expose underlying intimal connective tissue to platelets and other circulating factors. Further refinement of this hypothesis concluded endothelial cells may be chemically injured or activated and remain in place, while still stimulating an inflammatory response [12].

Two subsequent reports by the American Heart Association Council on Arteriosclerosis attempted to define the nature of the human arterial intima [13] as well as the features of the initial stages of atherosclerosis [14]. Adaptive or diffuse intimal thickening was described as a part of the normal intima and was shown to be present in a 16-month-old infant without evidence of lipid accumulation or other atherosclerotic changes. They also concluded that the locations of development of atherosclerotic lesions and of diffuse intimal thickening (DIT) are similar. The cellular makeup of the intimal thickening is primarily smooth muscle cells overlaid with endothelial cells and isolated macrophages. In initial lesions, foam cells were described as being macrophage in origin, though many of the studies, these conclusions were drawn from are animal models where DIT is lacking. Lipid deposition was seen in human arteries prior to the appearance of macrophage foam cells. The authors concluded that lesions occur preferentially in arteries with DIT and that the initial foam cells are macrophages; the definite identification of these cells as macrophages is uncertain given variable morphology of intimal SMCs and lack of antibodies at the time to specifically identify macrophages and may have been partly based on the assumption that foam cells must derive from macrophages, despite prior evidence that deep intimal foam cells appear to be SMC in origin. Many of the conclusions about initial foam cell formation appear to have been made using animal models that lack DIT, in which atherosclerotic lesions can only result from highly inflammatory conditions such as severe hyperlipidemia, and where monocyte/macrophages are recruited very early in atherogenesis. It does appear that in some reports of human lesions, macrophages compose the first foam cells, while the majority of reports suggested the initial foam cells are SMC derived. The consistent conclusion from these early studies however is that foam cells in humans develop in an intima already populated with a pre-atherosclerotic DIT layer composed almost entirely of SMCs and their secreted proteoglycans.

### Diffuse Intimal Thickening and Smooth Muscle Cell–Driven Retention of Lipoproteins Are the Initiators of Human Atherosclerosis

Diffuse intimal thickening is considered to be an adaptation to altered mechanical stress and occurs in regions where wall shear stress is reduced or wall tensile stress is elevated, or both [13]. Ikari et al. reported that population of the intima with SMCs to form DIT starts in utero with 36% of coronaries showing intimal thickening at birth; by age 2 years, all human subjects (91 cases) showed this process in the coronaries [3]. In the same study and other studies of normal arterial structure [15, 16•], no evidence of macrophage infiltration was found, suggesting DIT forms in the absence of any demonstrable level of injury to the endothelium, at least not enough to cause monocyte/macrophage infiltration [3]. DIT is routinely present in all arteries with risk of developing future atherosclerosis, consistent with it being a requirement for plaque development in humans [13, 15]. Current leading theories of the mechanism of development of DIT are that intimal SMCs likely derive from a subset of medial SMCs [17, 18•] and that the intima is populated following oligoclonal migration of medial SMCs and their proliferation in the intima, with only a subset of clones surviving [19].

**Fig. 2:**
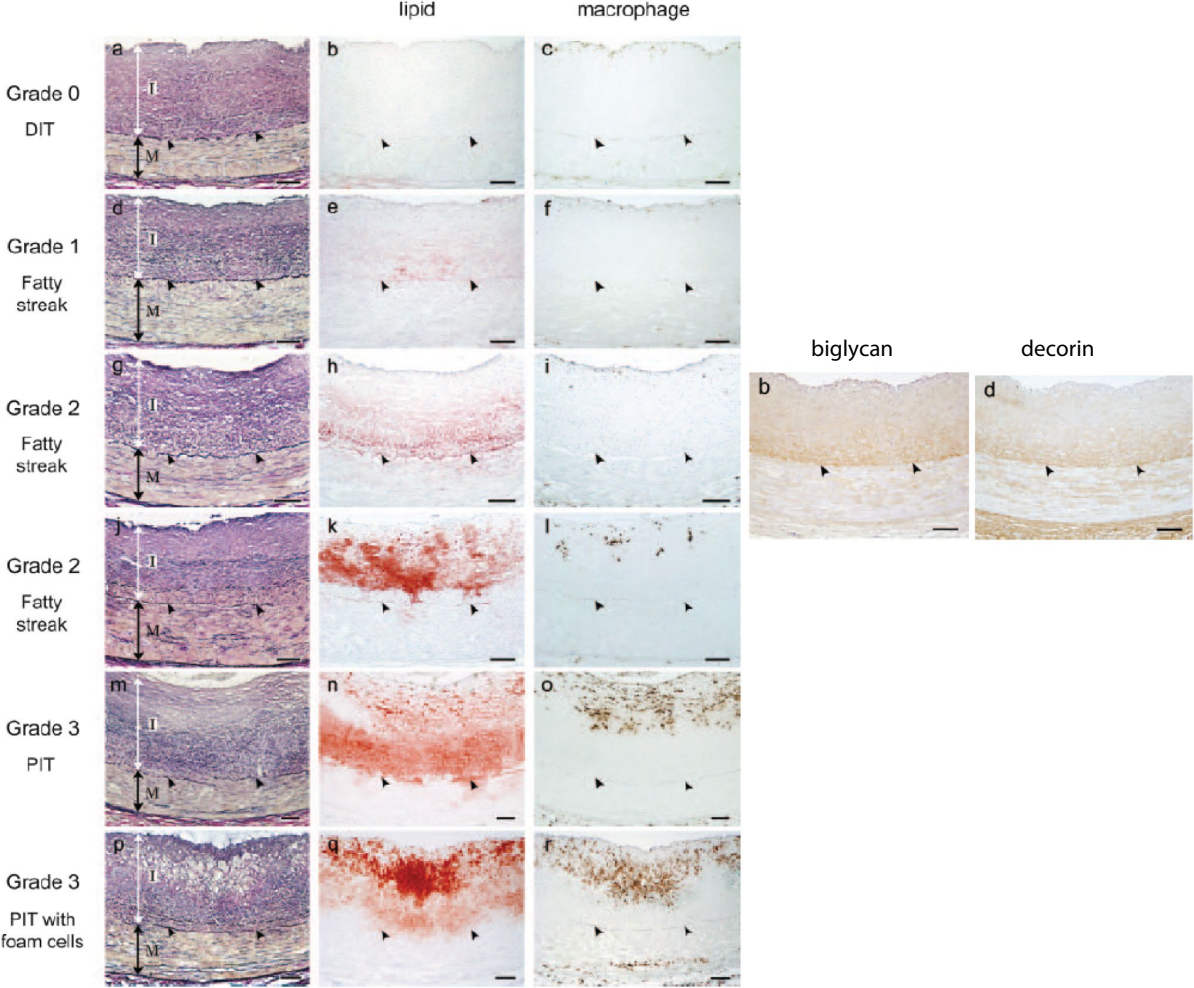
Progression of early atherosclerosis in human coronary arteries. The sections, from Nakashima et al. [16•], show the stages of atherosclerosis in human right coronary autopsy specimens from different subjects starting with pre-atherosclerotic diffuse intimal thickening (DIT, grade 0) prior to lipid or macrophage infiltration, deposition of lipids stained with Sudan IV in the deep intima in grade 1 and 2 fatty streaks prior to macrophage infiltration and in a similar location to highest expression of biglycan and decorin by deep intimal SMCs (right panels), and later macrophage accumulation as suggested by anti-CD68 staining in grade 3 lesions, pathological intimal thickening (PIT). Left column images were stained with elastica van Gieson. (I) intima; (M) media; arrowheads, internal elastic lamina; bar, 100 μm. (Reproduced from Arterioscler Thromb Vasc Biol. 2007 May; 27 [5]:1159–65, with permission from Wolters Kluwer Health, Inc.) [16•]

In their seminal 2007 paper, Nakashima and colleagues delineated clearly the progression of early atherosclerosis in humans from pre-atherosclerotic DIT to pathologic intimal thickening (Fig. 2) [16•]. Samples of the middle segment of the right coronary from 38 individuals ages 7 to 49 were studied, with average lipid levels in the subjects being in the normal range. As seen in the figure, a normal coronary artery of a 36-year-old male showed pre-atherosclerotic DIT (grade 0) with intimal thickness more than double medial thickness, no evidence of lipid deposition and a few scattered subendothelial macrophages. Early fatty streak formation (grade 1 and 2 lesions) was characterized by progressive deposition of lipids in the deep intima, in a pattern consistent with highest expression of SMC-derived biglycan and decorin also in this region (Fig. 2, right panels). This pattern is consistent with the 1995 conclusions of Williams and Tabas that atherosclerosis in humans is initiated by the retention of circulating lipoproteins containing positively charged apolipoprotein B binding to negatively charged proteoglycans in the intima [20]. In their paper, the authors concluded that this deposition can occur without any demonstrable evidence of endothelial injury or inflammation [2, 9, 14], as also evidenced in Nakashima et al. by the lack of any increase in macrophage infiltration at this stage [16•]. More recent studies have confirmed the primary mode of lipoprotein trafficking from the circulation across the endothelium to be transcytosis, an SR-BI and ALK1-dependent and LDL-receptor-independent process that does not require endothelial injury to occur [21•, 22•, 23•].

Further lipid deposition in grade 2 fatty streaks still clearly deposits around SMCs in the inner intima (Fig. 2), consistent with deep intimal SMCs being the initial source of foam cells within lesions as previously described [4–6, 9, 10]. SMCs express scavenger receptors like macrophages and also take up lipoproteins by pinocytosis [24–27]. Even a lesser capacity of SMCs to ingest lipoproteins compared to macrophages over time results in SMC foam cell formation, as the SMCs in the deep intima are bathed over decades in retained apoB-containing lipoproteins.

At the later fatty streak stage, there are still relatively few subendothelial macrophages. Only with later stage pathological intimal thickening do macrophages start to increase and penetrate deeper into the intima and participate in foam cell formation. The antibody used by Nakashima et al. to identify macrophages was against CD68, a marker now shown to also be expressed by intimal SMCs [28, 29]. As such, some of the cells identified as macrophage foam cells in this study are likely SMC derived. Clear differentiation between SMC and macrophage foam cells can only be achieved using antibodies against macrophage markers not known to be expressed by SMCs, such as CD45 [28, 30•, 31•].

This elegant study by Nakashima and colleagues and the preceding literature strongly indicate pre-lesional DIT composed almost entirely of SMCs and their secreted proteoglycans to be the necessary prerequisite for lipoprotein retention and development of atherosclerosis in humans that SMCs typically comprise the first lesion foam cells and that macrophages start to have a more significant role only following the fatty streak stage.

### The Role of Macrophages and SMCs in Mouse Atherosclerosis

Despite this extensive knowledge of the early pathogenesis of human atherosclerosis, the most common model of atherogenesis still presented is that it is driven by macrophages, as a response to endothelial injury, with little or no indicated role for SMCs in the initial stages of the disease [32–35]. This perception has been driven primarily by findings from animal models. Mice and other animal species that lack DIT cannot be made to develop atherosclerosis without inducing a highly inflammatory, usually hyperlipidemic state, as produced in mice by deletion of apolipoprotein E or the LDL receptor gene or overexpression of gain of function PSCK9 in combination with a high fat and cholesterol diet [36, 37]. Severe hyperlipidemia inflames the endothelium, resulting in high expression by endothelial cells of monocyte chemotactic factors and a rapid influx of monocytes along with lipoproteins into the intima as the initial stage of mouse atherosclerosis. Blocking initial monocyte migration into the intima and thereby maturation to macrophages can nearly halt the progression of atherosclerosis [38], confirming that macrophages are the key driver of initial atherogenesis in mice. The absence of SMCs in the healthy mouse intima is however quickly altered as chemokines secreted by inflamed endothelial cells and macrophages induce SMC migration from the medial layer into the intima [39]. SMCs in mouse atherosclerotic lesions undergo dedifferentiation, quickly exhibiting > 80% loss of classic SMC markers such as smooth muscle alpha actin and myosin heavy chain 11 [40] and activation of expression of macrophage markers such as CD68 and Mac-2 previously thought to be a macrophage/leukocyte specific [27, 40, 41]. This loss of SMC and gain of macrophage markers by mouse intimal SMCs has previously severely impeded identification and awareness of the roles of SMCs in mouse plaque. Identification of CD-45 as a leukocyte-specific marker not expressed by SMCs and the use of SMC-lineage tracing mice have led to a major advance in our ability to identify and quantitate SMCs in mouse lesions [28, 30•, 40]. In our study using apoE-deficient mice, following just 6 weeks of high-fat feeding, we determined that 70% of aortic arch foam cells in both male and female mice were CD-45 negative and subsequently confirmed them to be SMC-derived using SMC lineage-tracing mice [30•]. Thus, despite an intima that is completely devoid of SMCs at the disease outset, very rapidly, the mouse lesion is populated by proliferating SMCs that ingest intimal lipoproteins and soon become the major source of foam cells, rather than macrophages. These results have since been replicated in the PCSK9 gain of function model of LDL receptor deficiency, which found a similarly high percentage of mouse aortic foam cells derived from SMCs [31•].

### The Erroneous Concept of a SMC-Derived Macrophage

The loss of SMC markers and gain of macrophage marker proteins by mouse intimal SMCs [29, 40] has led to the impression that a population of SMCs can transition to becoming a reservoir of functional macrophages in lesions. The Fisher group showed that following cholesterol loading of cultured SMCs, and despite loss of SMC and gain of macrophage protein expression, SMCs exhibited only ~ 25% of the ability of macrophages to carry out phagocytosis and efferocytosis [41]. At best, SMCs can be said to be reprogrammed in the intima to a dysfunctional macrophage-like phenotype, but they never achieve ultimate functionality of macrophages [41]. In the same publication, the authors demonstrated that principal component analysis of the gene expression patterns of cholesterol-loaded SMCs remains closer to that of noncholesterol-loaded SMCs than to macrophages [41]. These findings indicate SMCs may partially but never fully transition to a true macrophage phenotype and that the concept of a SMC-derived macrophage is not supported by current literature.

### The Expanding Role of SMCs Within Atherosclerotic Plaque

The introduction of lineage-tracing mice in the last decade has greatly advanced our understanding of multiple previously unrecognized roles for SMCs in mouse atherosclerosis and how these findings can be applicable to the human disease [18•, 40, 42, 43•]. SMCs exhibit a high degree of plasticity [44•] and can reprogram in the lesion to assume various other phenotypes besides macrophage-like, including osteochondrogenic-like, myofibroblast-like, and mesenchymal stem cell-like [45]. Transcription factor Kruppl-like factor 4 (KLF4) has been shown to be a critical molecular switch for SMC differentiation from a contractile to a dedifferentiated transitional state [40, 46•]. The use of SMC-lineage tracing mice indicated > 60% of SMCs in atherosclerotic lesions dedifferentiate to a “pioneer” state that can transition to several multipotent states including macrophage-like and other forms [46•, 47]. In addition to potentially detrimental phenotypic transitions, Wirka et al. demonstrated transcription factor 21 (TCF21), promotes phenotypic modulation of SMCs to a fibromyocyte state that populates the protective fibrous cap and that increased expression of TCF21 is associated with decreased CAD risk in humans [48•]. SMC dedifferentiation can therefore be detrimental or potentially beneficial. The suitability of altering the course of differentiation of SMCs to these various states therapeutically is currently unknown but is an active area of research in the field [49•].

A recent publication reported that 60% (84 candidate causal genes) of known CAD-associated variants identified in genome-wide association studies show statistically significant expression quantitative trait locus (eQTL) or splicing QTL effects in vascular SMCs [50•]. A further study reported that 20% of eQTLs identifying candidate genes for the GWAS loci are specific to quiescent SMCs and another 35% specific to proliferating SMCs [51•]. These results indicate arterial SMCs of different phenotypes are responsible for a high degree of total CAD risk, as could be predicted based on their being by far the most abundant cell type in lesions.

One altered intimal SMC phenotype is the SMC foam cell, which, rather than being “macrophage-like,” exhibits distinctly different functional and phenotypic features compared to macrophage foam cells. We originally reported that intimal SMCs exhibit markedly lower expression of the key cholesterol exporter ABCA1 when compared to medial SMCs and proposed they should therefore constitute a large fraction of plaque foam cells [52]. Immunohistochemical studies indicated that, at minimum, SMCs contribute at least 50% of total foam cells in human coronary plaque and show reduced ABCA1 expression when compared to intimal macrophages [28]. Subsequent studies revealed that SMCs have markedly lower expression of *LIPA*, encoding lysosomal acid lipase (LAL), the sole lysosomal hydrolase capable of hydrolyzing lipoprotein-derived and intracellular cholesteryl esters, when compared to macrophages [53•]. Due to this relative LAL deficiency, SMCs retain lipoprotein-derived cholesteryl esters in lysosomes rather than storing excess cholesterol in cytosolic cholesteryl ester droplets the way macrophages do [53•]. As such, SMC foam cells not only contribute the majority of foam cells in human and mouse plaque [28, 30•, 31•], they represent a markedly different type of foam cell compared to macrophage foam cells. The lysosomal trapping of cholesteryl esters in SMCs may make these foam cells resistant to regression in plaque using currently existing lipid-lowering therapies. This represents one example whereby phenotypic changes in intimal SMCs represent a unique therapeutic target for the reduction of atherosclerosis [54].

### Macrophage Roles Relative to Smooth Muscle Cells in Human Atherosclerosis

A huge volume of work over the last 30 years has delineated the roles of macrophages in the initiation and progression of atherosclerosis. This work has by necessity been done primarily in mouse models, which as indicated require an extreme inflammatory, macrophage-driven state to allow atherogenesis. Macrophages participate in numerous pathways within the plaque including inflammation, apoptosis, necroptosis, phagocytosis, efferocytosis of dying and dead cells, clonal hematopoiesis, cholesterol efflux, and plaque regression [33, 55, 56]. The variable functions of macrophages contribute to plaque enlargement and instability but also to plaque stabilization and regression depending on the state of macrophage polarization and other factors including the level of circulating lipoproteins.

As indicated above, the role of macrophages in human atherosclerosis appears to be more important in intermediate to advanced stage lesions [16•], where macrophages are assumed to contribute in similar ways to the various pathways demonstrated in mice. The relative contribution of macrophages and SMCs to total plaque inflammation, stability or instability, and the ability of the plaque to regress are critical questions that require further investigation. The use of single-cell RNA sequencing of fresh human and mouse atheromas is allowing definitive analysis and comparison of the pathways and biologic itineraries of both macrophages and SMCs of varying phenotype [48•, 57–59]. Further studies including the use of scRNAseq and lesion foam cell analysis are required to determine the relative ability of macrophage and SMC foam cells to regress in the presence of lipid-lowering medications. Previously, it has been shown that in order for macrophage foam cells to leave the plaque, they must first release their excess lipids [60]. The relative inability of SMC foam cells to release excess cholesterol, due to their reduced expression of ABCA1 and LAL [28, 30•, 52, 53•], would predict that these cells are less likely to regress under the influence of intensive lipid-lowering therapy [61].

### Potential SMC-Targeted Therapies for Atherosclerosis

The fact that SMCs are the cell type contributing most to atherosclerotic lesions makes them a clear target to reduce the development and induce the regression of plaque. The migration of SMCs from the media to form the pre-atherosclerotic intima (DIT) occurs in all humans in fetal and early neonatal life, making it a challenging target for therapeutic intervention. Inhibiting the binding of apoB-containing lipoproteins to SMC-generated proteoglycans, for example by administering a vaccine to generate antibodies that block this interaction, represents the exciting possibility of using immunization to block lipoprotein retention in the artery wall lifelong for atherosclerosis prevention [62, 63]. The potential value of inhibiting dedifferentiation of SMCs within the plaque is unclear, given possible beneficial as well as harmful effects of less differentiated SMCs in lesions [48•]. The findings that SMC foam cells have impaired cholesterol efflux on the basis of reduced ABCA1 expression [28, 30•, 52], but that this can be at least partially overcome by delivering exogenous LAL to SMC foam cells [53•], represent an opportunity to specifically increase cholesterol efflux from SMC foam cells by increasing LAL delivery to these cells [53•, 54]. Interventions to increase cholesterol efflux from SMC foam cells have the potential to markedly improve upon the 30% relative reduction in major cardiovascular events achieved using currently available lipid-lowering therapies including statins, ezetimibe, and PCSK9 inhibitors [64], that may have their effect primarily on macrophage foam cells. Studies to address intervention in these and other specific aspects of intimal SMC biology are expected to uncover numerous novel targets for atherosclerosis prevention and treatment in the coming years.

## Conclusions

At this point, we are well beyond considering atherosclerosis to be a primarily macrophage-driven process, though it continues to be represented as such in many prominent reviews of this disease. Previous and recent data indicate that atherogenesis in humans is SMC-driven, based on the presence of pre-atherosclerotic diffuse intimal thickening composed almost entirely of SMCs and their secreted proteoglycans that drive initial lipoprotein retention in the artery wall. Evidence indicates SMCs represent the first foam cells formed in the lesion and contribute the majority of cholesterol-laden foam cells through later stages of atherosclerosis. Recent findings using macrophage-specific cell markers, SMC lineage-tracing mice, and GWAS CAD loci indicate a much larger role for SMCs in predicting lesion fate and overall CAD risk than previously known. Further studies will delineate the biologic itinerary of intimal SMCs and SMC foam cells and the suitability of SMCs to become a unique and novel therapeutic target to advance the prevention and treatment of ischemic atherosclerotic vascular disease.

## References

[1] Barquera S, Pedroza-Tobías A, Medina C, Hernández-Barrera L, Bibbins-Domingo K, Lozano R (2015). Global overview of the epidemiology of atherosclerotic cardiovascular disease. Arch Med Res..

[2] Wissler RW (1991). Update on the pathogenesis of atherosclerosis. Am J Med..

[3] Ikari Y, McManus BM, Kenyon J, Schwartz SM (1999). Neonatal intima formation in the human coronary artery. Arterioscler Thromb Vasc Biol..

[4] Virchow R. Cellular pathology [Internet]. London: John Churchill; 1858. https://archive.org/details/cellularpatholog00vircrich/mode/2up. Accessed 9 Jul 2023.

[5] Altschul R (1950). Selected studies on arteriosclerosis.

[6] Geer JC, McGILL HC, Strong JP (1961). The fine structure of human atherosclerotic lesions. Am J Pathol..

[7] Aqel NM, Ball RY, Waldmann H, Mitchinson MJ (1985). Identification of macrophages and smooth muscle cells in human atherosclerosis using monoclonal antibodies. J Pathol..

[8] Ross R, Wight TN, Strandness E, Thiele B (1984). Human atherosclerosis I. Cell constitution and characteristics of advanced lesions of the superficial femoral artery. Am J Pathol..

[9] Katsuda S, Boyd HC, Fligner C, Ross R, Gown AM (1992). Human atherosclerosis. III. Immunocytochemical analysis of the cell composition of lesions of young adults. Am J Pathol..

[10] Ross R, Glomset JA (1973). Atherosclerosis and the arterial smooth muscle cell: proliferation of smooth muscle is a key event in the genesis of the lesions of atherosclerosis. Science.

[11] Ross R, Glomset JA (1976). The pathogenesis of atherosclerosis (second of two parts). N Engl J Med..

[12] Ross R (1993). The pathogenesis of atherosclerosis: a perspective for the 1990s. Nature.

[13] Stary HC, Blankenhorn DH, Chandler AB, Glagov S, Insull W, Richardson M (1992). A definition of the intima of human arteries and of its atherosclerosis-prone regions. A report from the Committee on Vascular Lesions of the Council on Arteriosclerosis, American Heart Association. Circulation.

[14] Stary HC, Chandler AB, Glagov S, Guyton JR, Insull W, Rosenfeld ME (1994). A definition of initial, fatty streak, and intermediate lesions of atherosclerosis. A report from the Committee on Vascular Lesions of the Council on Arteriosclerosis, American Heart Association. Circulation.

[15] Nakashima Y, Chen YX, Kinukawa N, Sueishi K (2002). Distributions of diffuse intimal thickening in human arteries: preferential expression in atherosclerosis-prone arteries from an early age. Virchows Arch..

[16] Nakashima Y, Fujii H, Sumiyoshi S, Wight TN, Sueishi K (2007). Early human atherosclerosis: accumulation of lipid and proteoglycans in intimal thickenings followed by macrophage infiltration. Arterioscler Thromb Vasc Biol..

[17] Li S, Fan YS, Chow LH, Van Den Diepstraten C, van Der Veer E, Sims SM (2001). Innate diversity of adult human arterial smooth muscle cells: cloning of distinct subtypes from the internal thoracic artery. Circ Res..

[18] Chappell J, Harman JL, Narasimhan VM, Yu H, Foote K, Simons BD (2016). Extensive proliferation of a subset of differentiated, yet plastic, medial vascular smooth muscle cells contributes to neointimal formation in mouse injury and atherosclerosis models. Circ Res..

[19] Gomez D, Owens GK (2016). Reconciling smooth muscle cell oligoclonality and proliferative capacity in experimental atherosclerosis. Circ Res..

[20] Williams KJ, Tabas I (1995). The response-to-retention hypothesis of early atherogenesis. Arterioscler Thromb Vasc Biol..

[21] Kraehling JR, Chidlow JH, Rajagopal C, Sugiyama MG, Fowler JW, Lee MY (2016). Genome-wide RNAi screen reveals ALK1 mediates LDL uptake and transcytosis in endothelial cells. Nat Commun..

[22] Zhang X, Sessa WC, Fernández-Hernando C (2018). Endothelial transcytosis of lipoproteins in atherosclerosis. Front Cardiovasc Med..

[23] Huang L, Chambliss KL, Gao X, Yuhanna IS, Behling-Kelly E, Bergaya S (2019). SR-B1 drives endothelial cell LDL transcytosis via DOCK4 to promote atherosclerosis. Nature.

[24] Mietus-Snyder M, Gowri MS, Pitas RE (2000). Class A scavenger receptor up-regulation in smooth muscle cells by oxidized low density lipoprotein. Enhancement by calcium flux and concurrent cyclooxygenase-2 up-regulation. J Biol Chem..

[25] Llorente-Cortés V, Otero-Viñas M, Camino-López S, Costales P, Badimon L (2006). Cholesteryl esters of aggregated LDL are internalized by selective uptake in human vascular smooth muscle cells. Arterioscler Thromb Vasc Biol..

[26] Chellan B, Reardon CA, Getz GS, Hofmann Bowman MA (2016). Enzymatically modified low-density lipoprotein promotes foam cell formation in smooth muscle cells via macropinocytosis and enhances receptor-mediated uptake of oxidized low-density lipoprotein. Arterioscler Thromb Vasc Biol..

[27] Rong JX, Shapiro M, Trogan E, Fisher EA (2003). Transdifferentiation of mouse aortic smooth muscle cells to a macrophage-like state after cholesterol loading. Proc Natl Acad Sci U S A..

[28] Allahverdian S, Chehroudi AC, McManus BM, Abraham T, Francis GA (2014). Contribution of intimal smooth muscle cells to cholesterol accumulation and macrophage-like cells in human atherosclerosis. Circulation.

[29] Feil S, Fehrenbacher B, Lukowski R, Essmann F, Schulze-Osthoff K, Schaller M (2014). Transdifferentiation of vascular smooth muscle cells to macrophage-like cells during atherogenesis. Circ Res..

[30] Wang Y, Dubland JA, Allahverdian S, Asonye E, Sahin B, Jaw JE (2019). Smooth muscle cells contribute the majority of foam cells in ApoE (Apolipoprotein E)-deficient mouse atherosclerosis. Arterioscler Thromb Vasc Biol..

[31] Robichaud S, Rasheed A, Pietrangelo A, Doyoung Kim A, Boucher DM, Emerton C (2022). Autophagy is differentially regulated in leukocyte and nonleukocyte foam cells during atherosclerosis. Circ Res..

[32] Moore KJ, Sheedy FJ, Fisher EA (2013). Macrophages in atherosclerosis: a dynamic balance. Nat Rev Immunol..

[33] Libby P (2021). The changing landscape of atherosclerosis. Nature.

[34] Soehnlein O, Libby P (2021). Targeting inflammation in atherosclerosis - from experimental insights to the clinic. Nat Rev Drug Discov..

[35] Björkegren JLM, Lusis AJ (2022). Atherosclerosis: recent developments. Cell.

[36] von Scheidt M, Zhao Y, Kurt Z, Pan C, Zeng L, Yang X (2017). Applications and limitations of mouse models for understanding human atherosclerosis. Cell Metab..

[37] Zhao Y, Qu H, Wang Y, Xiao W, Zhang Y, Shi D (2020). Small rodent models of atherosclerosis. Biomed Pharmacother..

[38] Combadière C, Potteaux S, Rodero M, Simon T, Pezard A, Esposito B (2008). Combined inhibition of CCL2, CX3CR1, and CCR5 abrogates Ly6C(hi) and Ly6C(lo) monocytosis and almost abolishes atherosclerosis in hypercholesterolemic mice. Circulation.

[39] Ramji DP, Davies TS (2015). Cytokines in atherosclerosis: key players in all stages of disease and promising therapeutic targets. Cytokine Growth Factor Rev..

[40] Shankman LS, Gomez D, Cherepanova OA, Salmon M, Alencar GF, Haskins RM (2015). KLF4-dependent phenotypic modulation of smooth muscle cells has a key role in atherosclerotic plaque pathogenesis. Nat Med..

[41] Vengrenyuk Y, Nishi H, Long X, Ouimet M, Savji N, Martinez FO (2015). Cholesterol loading reprograms the microRNA-143/145-myocardin axis to convert aortic smooth muscle cells to a dysfunctional macrophage-like phenotype. Arterioscler Thromb Vasc Biol..

[42] Misra A, Feng Z, Chandran RR, Kabir I, Rotllan N, Aryal B (2018). Integrin beta3 regulates clonality and fate of smooth muscle-derived atherosclerotic plaque cells. Nat Commun..

[43] Dobnikar L, Taylor AL, Chappell J, Oldach P, Harman JL, Oerton E (2018). Disease-relevant transcriptional signatures identified in individual smooth muscle cells from healthy mouse vessels. Nat Commun..

[44] Allahverdian S, Chaabane C, Boukais K, Francis GA, Bochaton-Piallat ML (2018). Smooth muscle cell fate and plasticity in atherosclerosis. Cardiovasc Res..

[45] Grootaert MOJ, Bennett MR (2021). Vascular smooth muscle cells in atherosclerosis: time for a re-assessment. Cardiovasc Res..

[46] Alencar GF, Owsiany KM, Karnewar S, Sukhavasi K, Mocci G, Nguyen AT (2020). The stem cell pluripotency genes Klf4 and Oct4 regulate complex SMC phenotypic changes critical in late-stage atherosclerotic lesion pathogenesis. Circulation.

[47] Miano JM, Fisher EA, Majesky MW (2021). Fate and state of vascular smooth muscle cells in atherosclerosis. Circulation.

[48] Wirka RC, Wagh D, Paik DT, Pjanic M, Nguyen T, Miller CL (2019). Atheroprotective roles of smooth muscle cell phenotypic modulation and the TCF21 disease gene as revealed by single-cell analysis. Nat Med..

[49] Basatemur GL, Jørgensen HF, MCH C, Bennett MR, Mallat Z (2019). Vascular smooth muscle cells in atherosclerosis. Nat Rev Cardiol..

[50] Solomon CU, DG MV, Andreadi C, Gong P, Turner L, Stanczyk PJ (2022). Effects of coronary artery disease-associated variants on vascular smooth muscle cells. Circulation.

[51] Aherrahrou R, Lue D, Perry RN, Aberra YT, Khan MD, Soh JY (2023). Genetic regulation of SMC gene expression and splicing predict causal CAD genes. Circ Res..

[52] Choi HY, Rahmani M, Wong BW, Allahverdian S, McManus BM, Pickering JG (2009). ATP-binding cassette transporter A1 expression and apolipoprotein A-I binding are impaired in intima-type arterial smooth muscle cells. Circulation.

[53] Dubland JA, Allahverdian S, Besler KJ, Ortega C, Wang Y, Pryma CS (2021). Low LAL (lysosomal acid lipase) expression by smooth muscle cells relative to macrophages as a mechanism for arterial foam cell formation. Arterioscler Thromb Vasc Biol..

[54] Besler KJ, Blanchard V, Francis GA (2022). Lysosomal acid lipase deficiency: a rare inherited dyslipidemia but potential ubiquitous factor in the development of atherosclerosis and fatty liver disease. Front Genet..

[55] Farahi L, Sinha SK, Lusis AJ (2021). Roles of macrophages in atherogenesis. Front Pharmacol..

[56] Fidler TP, Xue C, Yalcinkaya M, Hardaway B, Abramowicz S, Xiao T (2021). The AIM2 inflammasome exacerbates atherosclerosis in clonal haematopoiesis. Nature.

[57] Slenders L, Landsmeer LPL, Cui K, Depuydt MAC, Verwer M, Mekke J (2022). Intersecting single-cell transcriptomics and genome-wide association studies identifies crucial cell populations and candidate genes for atherosclerosis. Eur Heart J Open..

[58] Abplanalp WT, Tucker N, Dimmeler S (2022). Single-cell technologies to decipher cardiovascular diseases. Eur Heart J..

[59] Ma WF, Hodonsky CJ, Turner AW, Wong D, Song Y, Mosquera JV (2022). Enhanced single-cell RNA-seq workflow reveals coronary artery disease cellular cross-talk and candidate drug targets. Atherosclerosis.

[60] Feig JE, Rong JX, Shamir R, Sanson M, Vengrenyuk Y, Liu J (2011). HDL promotes rapid atherosclerosis regression in mice and alters inflammatory properties of plaque monocyte-derived cells. Proc Natl Acad Sci U S A..

[61] Dubland JA, Francis GA (2016). So much cholesterol: the unrecognized importance of smooth muscle cells in atherosclerotic foam cell formation. Curr Opin Lipidol..

[62] Soto Y, Acosta E, Delgado L, Pérez A, Falcón V, Bécquer MA (2012). Antiatherosclerotic effect of an antibody that binds to extracellular matrix glycosaminoglycans. Arterioscler Thromb Vasc Biol..

[63] Allahverdian S, Ortega C, Francis GA (2022). Smooth muscle cell-proteoglycan-lipoprotein interactions as drivers of atherosclerosis. Handb Exp Pharmacol..

[64] Wang N, Woodward M, Huffman MD, Rodgers A (2022). Compounding benefits of cholesterol-lowering therapy for the reduction of major cardiovascular events: systematic review and meta-analysis. Circ Cardiovasc Qual Outcomes..

